# Anxiety severity and cognitive function in primary care patients with anxiety disorder: a cross-sectional study

**DOI:** 10.1186/s12888-021-03618-z

**Published:** 2021-12-09

**Authors:** Jenny Nyberg, Malin Henriksson, Alexander Wall, Torbjörn Vestberg, Maria Westerlund, Marion Walser, Robert Eggertsen, Louise Danielsson, H. Georg Kuhn, N. David Åberg, Margda Waern, Maria Åberg

**Affiliations:** 1 Section for Clinical Neuroscience, Institute of Neuroscience and Physiology, Sahlgrenska Academy, University of Gothenburg, Box 436, SE-405 30 Gothenburg, Sweden; 2 Region Västra Götaland, Neurology Clinic, Sahlgrenska University Hospital, SE-413 45 Gothenburg, Sweden; 3 School of Public Health and Community Medicine/Primary Health Care, Institute of Medicine, Sahlgrenska Academy, University of Gothenburg, Box 454, SE-405 30 Gothenburg, Sweden; 4 Department of Internal Medicine, Institute of Medicine, Sahlgrenska Academy, University of Gothenburg, Box 428, SE-405 30 Gothenburg, Sweden; 5 Region Västra Götaland, Department of Acute Medicine and Geriatrics, Sahlgrenska University Hospital, SE-413 45 Gothenburg, Sweden; 6 Department of Clinical Neuroscience, Karolinska Institutet, K8 Clinical Neuroscience, K8 Neuro Ingvar, SE-171 77 Stockholm, Sweden; 7 Region Västra Götaland, Regionhälsan, Gothenburg, Sweden; 8 R&D Centre Gothenburg and Södra Bohuslän, Kungsgatan 12, SE-411 19 Gothenburg, Sweden; 9 Department of Health and Rehabilitation, Institute of Neuroscience and Physiology, Sahlgrenska Academy, University of Gothenburg, Box 455, SE-405 30 Gothenburg, Sweden; 10 Region Västra Götaland, Angered Hospital, Box 63, SE-424 22 Gothenburg, Angered Sweden; 11 Department of Psychiatry and Neurochemistry, Institute of Neuroscience and Physiology, Sahlgrenska Academy, University of Gothenburg, Box 436, SE-405 30 Gothenburg, Sweden; 12 Region Västra Götaland, Psychosis Clinic, Sahlgrenska University Hospital, Mölndalsvägen 31 hus V, SE-431 80 Gothenburg, Sweden

**Keywords:** Anxiety disorder, Executive function, Cognitive function, Primary care, Wechsler adult intelligence scale

## Abstract

**Background:**

Deficits in cognitive performance are reported in patients with anxiety disorders, but research is limited and inconsistent. We aimed to investigate cross-sectional associations between cognitive function, with focus on executive function, and anxiety severity in primary care patients diagnosed with anxiety disorders.

**Methods:**

189 Swedish patients aged 18–65 years (31% men) with anxiety disorders diagnosed according to Mini International Neuropsychiatric Interview were included. Severity of anxiety was assessed using Beck Anxiety Inventory self-assessment scale. Digit span, block design and matrix reasoning tests from the Wechsler Adult Intelligence Scale IV, and the design fluency test from the Delis-Kaplan Executive Function System were used. Multivariable linear regression models were applied to investigate the relationship of anxiety severity and cognitive functioning. Comparisons were also performed to a normed non-clinical population, using the Wilcoxon signed rank test.

**Results:**

More severe anxiety was associated with lower digit span test scores (R^2^ = 0.109, B = -0.040, *p* = 0.018), but not with block design, matrix reasoning or design fluency tests scores, after adjustment for comorbid major depression in a multivariable model. When compared to a normed population, patients with anxiety performed significantly lower on the block design, digit span forward, digit span sequencing and matrix reasoning tests.

**Conclusions:**

Severity of anxiety among patients with anxiety disorder was associated with executive functions related to working memory, independently of comorbid major depression, but not with lower fluid intelligence. A further understanding of the executive behavioral control in patients with anxiety could allow for more tailored treatment strategies including medication, therapy and interventions targeted to improve specific cognitive domains.

**Supplementary Information:**

The online version contains supplementary material available at 10.1186/s12888-021-03618-z.

## Background

Anxiety disorders were ranked as the second leading cause of disease burden among all mental disorders, according to a recent Global Burden Disease report [1]. In addition to reducing quality of life and daily functioning [2], anxiety disorders are associated with elevated risks of cardiovascular disease [3] and premature mortality [4]. Deficits in cognitive performance have been reported, but findings are heterogeneous and inconsistent and few studies are set in the context of primary care [5–10]. There is some evidence that impairments in executive function (EF) may be of particular importance in the association between anxiety disorders and cognitive function [11].

EF refers to several “top-down”, effortful cognitive processes needed to regulate thoughts and actions during goal-directed behaviours [12, 13]. It is a complex cognitive concept including attention, inhibition, working memory (WM), cognitive flexibility, reasoning and problem-solving [12]. Attention is the ability to selectively attend and focus on a task at hand [12]. Inhibition, closely related to attention, involves being able to control one’s attention, behavior, thoughts, and/or emotions to override or suppress attention to other stimuli, and instead do what’s more appropriate or needed [12]. Deficits in attention and inhibition have been reported in patients with panic disorder (PD) and generalized anxiety disorder (GAD) [6, 14–16], though findings are inconsistent [9, 10]. WM involves holding information in mind (maintaining) and mentally working with it (manipulation) across a shorter delay [12]. Impairments in WM performance have been shown in patients with GAD [7] and in induced anxiety in a non-clinical population [17]. The evidence is mixed regarding PD, with WM deficiencies shown in one study [18] but not in others [10, 19]. Cognitive flexibility is being able to change perspectives spatially or interpersonally, ability to change how we think about something (think outside of the box) and flexibility to adjust to changed demands, to admit you were wrong, and to take advantage of sudden, unexpected opportunities [12]. Research on cognitive flexibility in persons with anxiety disorders is divided with studies showing impairments in patients with PD or GAD [6, 15, 18], but also lack of such impairments [6, 19].

EF is also highly correlated to dimensions of the concept fluid intelligence i.e. the ability to understand relationships among components, to reason and solve problems [12, 20]. We could not find any studies specifically analysing fluid intelligence in patients with anxiety disorders in adults, but a lack of an association of fluid intelligence and anxiety disorders (excluding specific phobia) has been shown in adolescents [21]. Research on potential associations between anxiety severity and EF are scarce, but one study reported an inverse association between symptom severity and EF in patients with social anxiety disorder (SAD) [8]. Associations with other anxiety disorders remain to be clarified.

Associations between anxiety disorders and EF are important to study as deficits in EF may reduce a patient’s coping abilities, affecting a patient’s ability to function socially and occupationally in everyday life [11]. There are also clinical implications including enhanced screening and better understanding of treatment mechanisms [11]. When investigating the relationship between anxiety and cognitive functions, it is important to also consider comorbid depression. Presence of comorbid depression may confound the results as previous studies have demonstrated an association of depression and impaired functions within the domains of EF [22, 23]. Moreover, comorbid anxiety and depression might also represent a more severe illness state, than anxiety alone [24].

It has been estimated that 70% of individuals seeking help for anxiety initially present in primary care [25] and costs for mental illness within Swedish primary care are on the rise [26]. However, the relationship between anxiety disorders and cognitive performance has been little studied in this setting. The primary aim was to investigate whether level of EF was cross-sectionally associated with severity of anxiety in patients diagnosed with anxiety disorders (PD, GAD and anxiety not otherwise specified), in a primary care setting. Association of fluid intelligence and anxiety severity was also investigated due to the high correlation of fluid intelligence and the EFs problem-solving and reasoning [12]. Our hypothesis was that functions attributed to EFs (WM, inhibition, cognitive flexibility and attention) and fluid intelligence would be inversely associated with anxiety severity [7, 8, 27]. Secondary aims were to investigate these associations also in models adjusted for the existence of depression and to analyse EF performance in patients diagnosed with the anxiety disorders specified above in relation to a normed population.

## Methods

### Participants and settings

Participants for this cross-sectional study originate from the ongoing randomized controlled study Swedish Physical Fitness and Brain - Interventional Study (PHYSBI; NCT03247270; Trial Registration Date: 08/08/2017), focusing on investigating effects of an exercise intervention on symptoms of anxiety and cognitive function in patients with anxiety disorders [28]. Individuals who sought help for anxiety issues at six primary care units in Gothenburg (Närhälsan Primary Care) and Region Halland were recruited. Potential participants were diagnosed by a study psychiatrist using the Mini International Neuropsychiatric Interview (M.I.N.I; Swedish version 7.0.0 DSM 5), a structured diagnostic interview with high reliability and validity [29]. Patients aged 18–65 were included if diagnosed with the anxiety disorders PD (DSM 300.01) or GAD (DSM 300.02) according to M.I.N.I. In addition, patients with anxiety not otherwise specified (NOS; DSM 300.00) were also included after being diagnosed by the study psychiatrist. In order to maintain statistical power for the analyses, patients with aforementioned anxiety disorders were grouped together and denoted as patients with anxiety disorders. Patients with and without ongoing treatment with psychotropic medication were included. Individuals with ongoing psychotherapy were excluded since psychotherapy was viewed as a “commitment” in terms of time and energy which could impact adherence to the exercise intervention. Additional exclusion criteria included high suicide risk (patients with low to moderate suicide risk were included) or serious neurodevelopmental or psychotic disorders (milder cases were included) as assessed by the study general practitioner (GP). Pregnant women were not included in the current study. The study was approved by the Regional Ethics Committee in the Gothenburg, Sweden and was carried out in accordance with the Declaration of Helsinki (2013). Each participant signed a statement of informed consent after the nature of the procedures had been fully explained. For further details regarding the study methodology including sample size calculations, please see the study protocol [28].

### Assessment of anxiety severity

Severity of perceived ongoing symptoms of anxiety at baseline was self-assessed using the Swedish version (©2005 by NCS Pearson) of the clinically well-established Beck Anxiety Inventory (BAI) [30]. BAI mainly evaluates somatic symptoms and was developed to be relatively free from contamination by depressive content [30]. Both reliability [31] and validity [32] are reported to be good.

### Cognitive tests

Cognitive performance was measured using Wechsler Adult Intelligence Scale 4th edition (WAIS-IV) and the Delis-Kaplan Executive Function System (D-KEFS). The cognitive tests were applied by a licensed psychologist. WAIS-IV is a battery of tests measuring intelligence and cognitive functions, standardized on a normative sample of individuals (ages 16–90) and stratified to match a Scandinavian population based on age, sex, education, ethnicity and geographic region. In the current study, we used the block design, digit span and matrix reasoning tests including scaled scores from 1 to 20 with a normed mean of 10 and standard deviation (SD) of 3 [33]. Full WAIS-IV assessment was not employed due to the long completion time. For full information including WAIS-IV test descriptions, subtest modifications and reliability/validity statistics, see the WAIS-IV technical manual [33].

The Delis-Kaplan Executive Function System (D-KEFS) is a standardized, non-verbal psychomotor test battery aimed at assessing EFs in individuals aged between 8 and 89 years [34]. D-KEFS has been used in both clinical and research settings showing good reliability and validity for measuring EF [35]. The D-KEFS normative sample was composed using the 2000 U.S. Census figures as target values [35, 36]. The sample included over 1700 children, adolescents and adults (ages 8 to 89 years) and was based on demographic characteristics (including age, gender, socioeconomic factors) of the U.S. population [34]. Since the normative samples were divided into age groups, every patient tested using D-KEFS is compared to a large group of individuals of the same age span. WAIS-IV tests were performed before D-KEFS in all participants.

#### Block design

Subjects had to replicate red and white pattern designs using three-dimensional coloured blocks. This test measures functions including visual perception and problem-solving and non-verbal reasoning [33]. The block design test is commonly used as a measure of fluid intelligence [37–39], but it involves reasoning and problem-solving which are functions also attributed to EF [12].

#### Digit span

Subjects were asked to repeat a sequence of numbers read to them in order (forward), in reverse order (backward) or in ascending order of magnitude (sequencing). Digit span taps into functions including WM, attention, encoding and auditory processing. Digit span forward primarily measures short-term memory and attention [12, 40], digit span backward measures WM and digit span sequencing captures functions such as cognitive flexibility [33]. A combined score for all three digit span subtests (included in the WM cognitive domain) [33], was also obtained.

#### Matrix reasoning

Subjects had to solve a task presented in a visual format and identify patterns in designs. This test includes perceptual reasoning, non-verbal problem-solving and visuospatial ability [33], and is usually used as a measure of fluid intelligence. However, as for the block design test, the matrix reasoning test also involves reasoning and problem-solving which also are part of EF.

#### Design fluency

In the current study, we used the D-KEFS design fluency test in order to assess the ability to generate a series of novel (non-repeating) and abstract designs. A rationale for choosing this test is that it simulates the cognitive chain required in daily life to generate novel responses, while maintaining focus on a desired goal [34, 35], and involves multiple EFs including creativity, attention, inhibition and scanning and cognitive flexibility to find novel solutions.

The design fluency test is performed using a pen and paper and consists of three conditions of increasing difficulty where the subject has to connect dots and make novel shapes within a time limit [12, 34]. In the first condition, the subject had to create novel patterns by combining filled dots with four lines without repeating previous combinations, which demands creativity in drawing new designs. The second condition was the same as the first, but now with unfilled dots instead which increases the demand also for inhibition. The third condition consists of both filled and unfilled dots where the subject had to switch between filled and unfilled dots when creating the patterns, and which adds the requirement for cognitive flexibility. The total number of correct patterns and number of total patterns within the 60 s time limit was recorded for each condition and raw scores from each subtest were converted to age-adjusted scaled scores ranging from 1 to 19 (mean 10; SD 3), before analysis [34].

### Other measures

Comorbid psychiatric disorders including major depression were diagnosed by a psychiatrist using the M.I.N.I. Severity of ongoing symptoms of depression were self-assessed with the Montgomery Åsberg Depression Rating Scale (MADRS-S) [41]. The following variables were self-reported by study participants in a questionnaire designed by the research team: marital status, education level, years with anxiety symptoms, smoking and ongoing use of prescribed antidepressants (ATC N06) and psycholeptics/anitiepileptics (ATC N05 and N03).

### Procedure

Patients were recruited at five different time points from August 2017 to September 2019. The patients were informed by their GP or primary care psychologist about the possibility of study participation and those expressing interest were contacted by the study physician for further information. Diagnoses and comorbidities were determined by a psychiatrist and the cognitive tests were administered by a psychologist.

### Statistical analyses

All analyses were performed using Statistical Package for the Social Sciences (SPSS), 25.0 software (SPSS Inc., Chicago, IL). Characteristics of the whole study group are presented using descriptive statistics including number of observations, means and SD for continuous variables. Frequencies and percentages are presented for categorical variables.

Additional analyses were performed comparing characteristics, including performance on cognitive tests, of patients with or without psychotropic medication (antidepressant and/or psycholeptics/anitiepileptics), as well as patients with minimal/mild vs. medium/severe anxiety. For these, Pearson’s χ^2^-test was used for categorical variables and Mann-Whitney U-test was used for continuous variables. Normality was assessed graphically, and for most variables there were skewed distributions, and hence the Mann-Whitney U-test was used.

In order to investigate the relationship between severity of anxiety and cognitive functioning, multiple linear regression analyses were performed with self-reported BAI scores as an independent, continuous variable and performance scores on block design, digit span, matrix reasoning and design fluency tests as dependent variables. Different multivariable models were analysed including age, gender [42], smoking [43–45], education level and comorbid major depression (assessed through M.I.N.I.). Additional regression analyses were performed comparing patients with minimal/mild (BAI 0–16) and medium/severe anxiety (BAI 17–63) [46].

To compare cognitive functioning in patients with anxiety disorder to an age-adjusted normed population, the Wilcoxon signed rank test with standard algorithms was used (with a normed mean of 10, and an SD of 3 for all tests) [33, 35]. These analyses were performed since the current study design did not involve a group of persons without anxiety disorders for comparison. This method has been used previously for measuring cognitive function in young men [47]. For these we calculated N-1, which represents the degrees of freedom, and t-values from the F(df_regression_,df_residual_) = F_regression_ eq. *P*-values < 0.05 were considered statistically significant.

## Results

### Characteristics

In total, 189 patients were included in the current study after screening. See Table 1 for baseline characteristics of the sample. Over two thirds of the participants were women and the mean age was 39 years. The two most common diagnoses were PD (53%) and GAD (57%) and comorbid conditions were common; 42% had comorbid major depression. Two thirds of the study participants were on antidepressant and/or psycholeptic/anitiepileptic medication, mainly antidepressants. Mean self-rated anxiety BAI score was 24.8 (SD 12.7), which corresponds to moderate/severe anxiety. Half of the participants (53%) reported onset of anxiety more than 10 years ago. Onset during the past five years was not as common (10%). Mean self-rated depression score (MADRS-S) was 21.7 (SD 8.2), corresponding to a moderate depression.

**Table 1:** Characteristics of study participants with anxiety disorders

Variables	Mean [SD] or N (%)	N
Age (years)	38.7 [12.2]	189
Men	59 (31.2)	189
Education above high school	104 (55.6)	187
Smoking	38 (20.2)	188
Psychotropic medication	126 (66.7)	189
Antidepressants^a^	101 (53.4)	189
Psycholeptics/anitiepileptics^b^	57 (30.2)	189
Unmarried	131 (70.8)	185
Married	54 (28.6)	185
**Rating Scales**		
BAI (score)	24.8 [12.7]	189
MADRS-S (score)	21.7 [8.2]	189
**WAIS-IV test scores**		
Block design	9.4 [3.1]	177
Digit span total	9.9 [2.8]	188
Digit span forward	9.0 [3.4]	177
Digit span backward	9.8 [2.7]	177
Digit span sequencing	9.4 [2.8]	177
Matrix reasoning	9.3 [2.9]	174
**D-KEFS design fluency test scores**		
Total correct designs	11.4 [2.9]	186
Correct designs condition 1	10.6 [2.8]	186
Correct designs condition 2	10.5 [2.7]	186
Correct designs condition 3	11.4 [2.7]	186
Total attempted designs	12.2 [3.5]	186
**Anxiety diagnoses**		
Panic disorder	100 (53.2)	188
Generalized anxiety disorder	107 (56.9)	188
Anxiety NOS	31 (16.5)	188
**Comorbidities**		
Major depression	78 (41.5)	188
Suicidality	43 (22.9)	188
Social phobia	76 (40.4)	188
Agoraphobia	64 (34.0)	188
Post-traumatic stress disorder	26 (13.8)	188
Alcohol use disorder	19 (10.1)	188
Substance use	4 (2.1)	188
Personality disorder (Antisocial)	19 (10.1)	188
Obsessive compulsive disorder	20 (10.6)	188
Bulimia nervosa	12 (6.4)	188

^a^ ATC N06

^b^ ATC N03 and N05

Values are given as means and standard deviations (SD) for continuous variables and frequencies with percentages for categorical variables along with the total number (N) of participants included

*D-KEFS* Delis–Kaplan Executive Function System, *MADRS-S* Montgomery Åsberg Depression Rating Scale Self-rated, *NOS* Not otherwise specified, *WAIS * Wechsler Adult Intelligence Scale

### Associations of anxiety severity and cognitive function

Significant regression equations were found for block design, digit span, matrix reasoning and design fluency condition 2 tests when adjusting for age and gender only (Model 1), where higher BAI scores were associated with lower test performance (Table 2). When also adjusting for smoking (Model 2), higher BAI scores no longer significantly associated with performance on the block design test. When adding education as an additional covariate (Model 3), significant associations were found for the digit span total (R^2^ = 0.109, B = -0.041, *p* = 0.011) and backward (R^2^ = 0.085, B = -0.042, p = 0.011) subtests and the matrix reasoning test (R^2^ = 0.134, B = -0.036, *p* = 0.035).

**Table 2:** Association of anxiety severity (BAI score) and cognitive function test scores

Cognitive function test scores	B	r	Equation	R^2^	*P* value
**Model 1**^a^					
WAIS-IV tests					
Block design	−0.045	0.352	F(3,170) = 8.019	0.109	**0.014**
Digit span total	−0.049	0.230	F(3,180) = 3.346	0.053	**0.003**
Digit span forward	−0.049	0.198	F(3,170) = 2.323	0.039	**0.017**
Digit span backward	−0.049	0.231	F(3,170) = 3.196	0.053	**0.003**
Digit span sequencing	−0.040	0.188	F(3,170) = 2.076	0.035	**0.021**
Matrix reasoning	−0.041	0.316	F(3,167) = 6.163	0.100	**0.015**
D-KEFS design fluency					
Total correct designs	−0.030	0.139	F(3,178) = 1.177	0.019	0.080
Correct designs condition 1	−0.024	0.161	F(3,178) = 1.580	0.026	0.149
Correct designs condition 2	−0.034	0.201	F(3,178) = 2.495	0.040	**0.032**
Correct designs condition 3	−0.020	0.120	F(3,178) = 0.862	0.014	0.219
Total attempted designs	−0.030	0.168	F(3,178) = 1.722	0.028	0.157
**Model 2**^b^					
WAIS-IV tests					
Block design	−0.035	0.406	F(4,169) = 8.361	0.145	0.054
Digit span total	−0.046	0.251	F(4,179) = 3.010	0.063	**0.006**
Digit span forward	−0.044	0.223	F(4,169) = 2.207	0.050	**0.036**
Digit span backward	−0.045	0.245	F(4,169) = 2.709	0.060	**0.007**
Digit span sequencing	−0.035	0.219	F(4,169) = 2.119	0.048	**0.046**
Matrix reasoning	−0.040	0.317	F(4,166) = 4.637	0.101	**0.021**
D-KEFS design fluency					
Total correct designs	− 0.029	0.141	F(4,177) = 0.903	0.020	0.093
Correct designs condition 1	− 0.022	0.175	F(4,177) = 1.399	0.031	0.199
Correct designs condition 2	−0.033	0.203	F(4,177) = 1.895	0.041	**0.039**
Correct designs condition 3	−0.021	0.121	F(4,177) = 0.656	0.015	0.213
Total attempted designs	−0.029	0.169	F(4,177) = 1.294	0.028	0.172
**Model 3**^c^					
WAIS-IV tests					
Block design	−0.030	0.456	F(5,168) = 8.813	0.184	0.093
Digit span total	−0.041	0.329	F(5,179) = 4.334	0.109	**0.011**
Digit span forward	−0.040	0.276	F(5,168) = 2.769	0.076	0.057
Digit span backward	−0.042	0.292	F(5,168) = 3.120	0.085	**0.011**
Digit span sequencing	−0.031	0.279	F(5,168) = 2.841	0.078	0.074
Matrix reasoning	−0.036	0.366	F(5,165) = 5.120	0.134	**0.035**
D-KEFS design fluency					
Total correct designs	−0.023	0.313	F(5,176) = 3.827	0.098	0.162
Correct designs condition 1	−0.016	0.326	F(5,176) = 4.200	0.107	0.321
Correct designs condition 2	−0.029	0.304	F(5,176) = 3.577	0.092	0.067
Correct designs condition 3	−0.017	0.237	F(5,176) = 2.091	0.056	0.306
Total attempted designs	−0.022	0.333	F(5,176) = 4.385	0.111	0.286

^a^ Age and gender as covariates

^b^ Smoking, age and gender as covariates

^c^ Education, smoking, age and gender as covariates

Statistically significant results (*p* < 0.05) indicated in bold

*BAI* Beck Anxiety Inventory, *D-KEFS* Delis–Kaplan Executive Function System, *WAIS* Wechsler Adult Intelligence Scale

In analyses with additional adjustment for comorbid major depression, BAI scores only associated with scores on digit span total (R^2^ = 0.109, B = -0.040, *p* = 0.018) and backward (R^2^ = 0.089, B = -0.045, *p* = 0.008) subtests, but not on the block design, matrix reasoning, design fluency tests or the digit span forward subtest (Table 3).

**Table 3:** Association of anxiety severity (BAI score) and cognitive function test scores, also adjusting for depression

Cognitive function test scores			B	r	Equation	R^2^	P value
WAIS-IV tests							
	Block design		−0.036	0.467	F(3,168) = 7.761	0.190	0.050
	Digit span total		−0.040	0.331	F(6,177) = 3.623	0.109	**0.018**
		Digit span forward	−0.038	0.277	F(6,167) = 2.316	0.077	0.076
		Digit span backward	−0.045	0.299	F(6,167) = 2.736	0.089	**0.008**
		Digit span sequencing	−0.030	0.280	F(6,167) = 2.363	0.078	0.091
	Matrix reasoning		−0.032	0.373	F(6,164) = 4.426	0.139	0.063
D-KEFS design fluency							
	Total correct designs		−0.019	0.322	F(6,175) = 3.367	0.103	0.261
		Correct designs condition 1	−0.014	0.330	F(6,175) = 3.555	0.109	0.413
		Correct designs condition 2	−0.028	0.305	F(6,175) = 2.988	0.093	0.090
		Correct designs condition 3	−0.011	0.258	F(6,175) = 2.076	0.066	0.499
	Total attempted designs		−0.020	0.334	F(6,175) = 3.653	0.111	0.337

Comorbid major depression, education, smoking, age and gender as covariates

Statistically significant results (*p* > 0.05) indicated in bold

*BAI* Beck Anxiety Inventory, *D-KEFS* Delis–Kaplan Executive Function System, *WAIS *Wechsler Adult Intelligence Scale

**Fig. 1:**
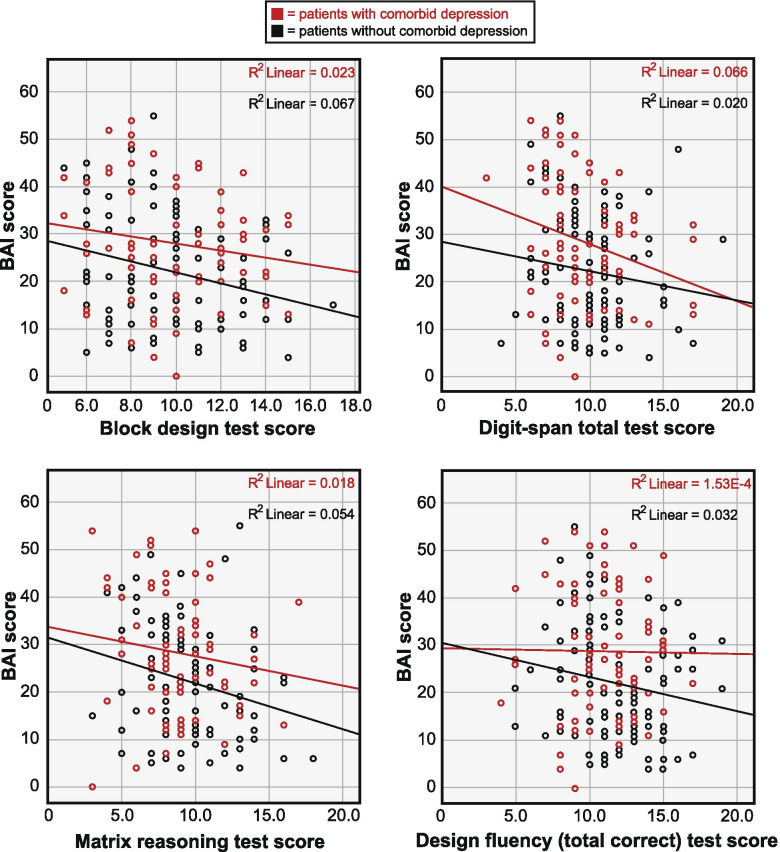
Scatterplots showing unadjusted relationships between Beck Anxiety Inventory score (BAI) and performance on the block design, digit span total, matrix reasoning test and design fluency (total correct designs) stratified separately for patients with (red, *n* = 78) and without (black, *n* = 110) comorbid major depression

Scatterplots for relationships between BAI scores and scores on the block design, digit span total, matrix reasoning test and design fluency (total correct designs) in patients with or without comorbid major depression are shown in Fig. 1. Frequency distributions of test scores for the block design, digit span, matrix reasoning and design fluency tests are shown in Additional file 1.

Additional analyses were performed comparing cognitive functioning in patients with (*n* = 126) and without (*n* = 63) ongoing medication with antidepressants and/or psycholeptics/anitiepileptics (Additional file 2). Scores on all cognitive tests did not differ between these two groups. Additional analyses comparing cognitive function of patients with minimal/mild anxiety (*n* = 61) to patients with moderate/severe anxiety (*n* = 128) showed that those with moderate/severe anxiety scored lower on digit span total (*p* = 0.016), forward (*p* = 0.042) and backward (*p* = 0.030) subtests (Additional file 3). No differences were observed in performance on the block design, matrix reasoning and design fluency tests.

### Cognitive function compared to a normed non-clinical population

**Fig. 2:**
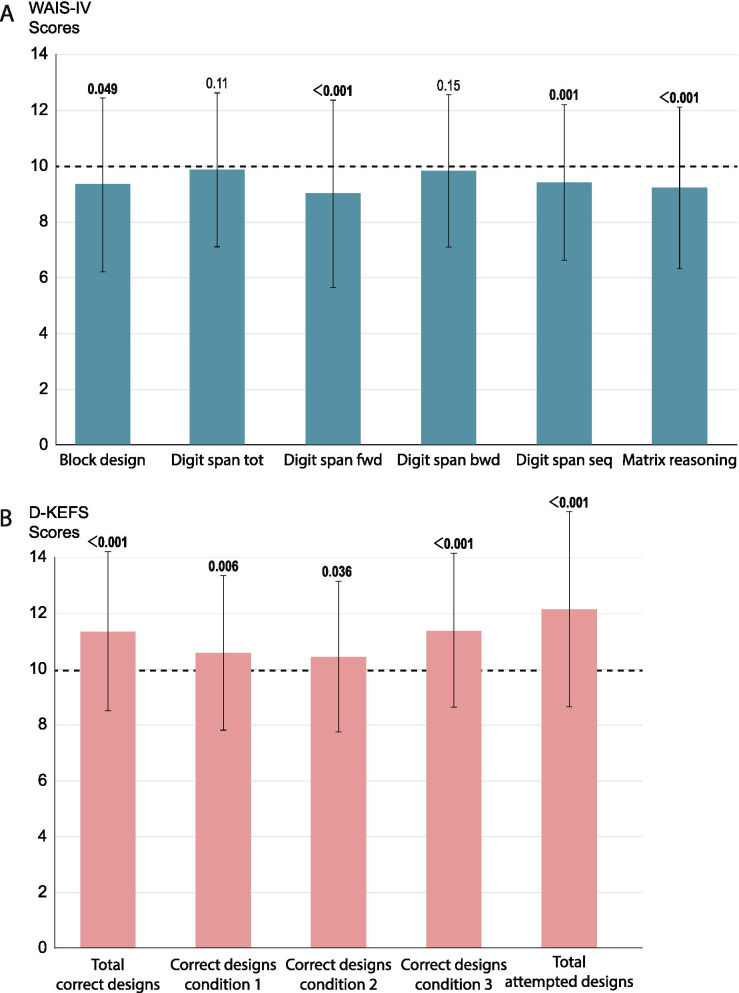
Performance scores on block design, digit span and matrix reasoning tests using WAIS-IV (A; *n* = 120) and design fluency test using D-KEFS (B; *n* = 126) in patients with anxiety disorder compared to a normed population (normed mean = 10; dashed line, standard deviation (SD) = 3, for all tests; see methods for significance testing). Error bars represent SD, *p*-values given above. Tot: total; fwd: forward; bwd: backward; seq: sequencing

Patients with anxiety disorder scored significantly lower on the block design, digit span forward and sequencing, and matrix reasoning tests than a normed population (Fig. 2 A). On the other hand, patients with anxiety disorder scored higher on all the design fluency tests (Fig. 2B). Full statistical information including means and SD are shown in Additional file 4.

## Discussion

In this cohort of primary care patients with anxiety disorders (PD, GAD and anxiety not otherwise specified), higher anxiety score was associated with lower EFs specifically related to WM (digit span) in multivariable models, after adjustment for comorbid major depression. This finding is also supported by our additional analyses showing that patients with moderate/severe anxiety symptoms scoring lower on the digit span test, compared to patients with minimal/mild symptoms.

The above result is in line with our hypothesis, that there would be a negative association of anxiety severity and measures of WM. In analyses adjusting for comorbid major depression, we observed an association of anxiety severity with scores on the digit span total and backward tests, but not with forward and sequencing tests. Digit span total is a general measure of WM and digit span backward specifically involves EF resources related to an active WM where information also is manipulated [12, 48]. Digit span forward on the other hand primarily measures a “passive” short-term memory where information is maintained [12] and digit span sequencing is a measure of cognitive flexibility. The inverse associations of anxiety severity and digit span total and backward scores in models adjusted for comorbid major depression, therefore indicate a relation between severity of anxiety and EFs related to WM, and not short-term memory or cognitive flexibility. Although we could find no studies investigating cross-sectional associations between anxiety level and WM performance for comparison, we note that patients with GAD (and without depression) scored lower on WM performance compared to healthy subjects [7]. This WM deficit was connected to lower prefrontal engagement; it was suggested to represent a key component of clinical anxiety, rather than a consequence of threat. On the other hand, in a review of PD and cognitive function, limited support was found for an association of PD alone in the absence of depression and WM [10]. Contrary to our hypothesis, we did not find an association between cognitive flexibility and anxiety severity. Lower cognitive flexibility have previously been reported for patients with PD with and without comorbid depression, but not for patients with GAD [6]. Our results might in part be explained by the large proportion of patients with GAD (57%) included in the current study. However, younger patients (20–30 years old) with GAD without comorbid depression have demonstrated impaired cognitive flexibility [15].

Although the block design and matrix reasoning tests are commonly used as measures of fluid intelligence, they also measure reasoning and problem-solving which are highly correlated to EFs. Our hypothesis was therefore that more severe anxiety would be associated with lower performance on these tests, but our results from analyses adjusted for comorbid major depression did not support our hypothesis. Lack of association of between anxiety symptoms and subsequent fluid intelligence (block design test) was also found in a study of Swedish twins (aged 50 or older), in models adjusted for depressive symptoms [49]. Lower fluid intelligence may hence be more related to the depressive state and not to anxiety, as also indicated by our multivariable models not adjusted for comorbid major depression. In line with the twin study, we conclude that severity of anxiety is not associated with fluid intelligence including the related EFs problem-solving and reasoning in the current study group of primary care patients.

We did not find an association between anxiety severity and performance on the design fluency tests (measuring aspects of EF including creativity, inhibition and cognitive flexibility) and could therefore not confirm our hypothesis regarding cognitive flexibility and inhibition. Lower design fluency scores have been shown in patients with comorbid clinical depression and anxiety [50]. However, absence of a dose-response relationship between number of comorbid anxiety disorders (as a measure of severity) and design fluency scores, after adjusting for comorbid depression, has also been reported in a large population-based sample of adults [24]. More research is clearly needed to investigate associations of EFs and severity of anxiety.

Psychotropic medication may influence cognitive function, which may affect the results [51, 52]. Antidepressants have been shown to have positive effects on EF in depressed patients [51], but it remains unclear whether this is the case in patients with anxiety disorders. We did not observe any between-group differences in cognitive performance in patients with or without medication, but confounding by indication must be taken into consideration.

### Cognitive function in patients with anxiety disorder compared to a normed population

Our study patients scored lower on tests on EFs related to WM and fluid intelligence compared to a normed population. Fluid intelligence is a wider concept including the ability to solve novel problems by using reasoning and not depending on accumulated knowledge such as schooling and acculturation [20]. Fluid intelligence might thus be a factor differentiating individuals with or without a clinical anxiety disorder, while WM deficits might only be obvious among diagnosed patients with more severe anxiety symptoms.

Surprisingly, our results showed that overall design fluency test performance of patients with anxiety were higher than the normed mean for the population. This result may be interpreted as patients with anxiety have better EF. There are sporadic reports of an association between anxiety disorders and “creativity” such as a creative occupation [24]. However, a more plausible explanation for our results may involve the normative sample for the design fluency test. The D-KEFS normative sample was composed using the 2000 U.S. Census figures as target values [35], but cross-national differences in cognitive function exist [53]. One possible explanation for our results is that Swedish D-KEFS means might be higher than U.S. means. WAIS-IV, on the other hand, was normed against a Scandinavian population [33]. Another possibility is the presence of a “Flynn effect” (the globally observed rise in intelligence test scores over time) since the construction of the D-KEFS normative sample in 2000 [54].

### Limitations

Several limitations are acknowledged. The cross-sectional design of the study excludes causal inferences between anxiety severity and EFs. Deficits in EF can be a consequence of, but may also increase vulnerability to the development and maintenance of anxiety disorder. The relationship may also be bidirectional. The causal link between anxiety disorder and EF has not been well established and longitudinal studies will be required to elucidate this relationship. The choice of measure for anxiety symptoms might impact the results. We chose to use BAI since it is well established within Swedish primary care and minimizes influences of depressive content. Although BAI was developed to capture both somatic and cognitive aspects of anxiety, it focuses primarily on somatic symptoms (Beck 1998). A different measure such as the commonly used State-Trait Anxiety Inventory (STAI) might yield different results. However, STAI was not the most appropriate for the current study given that the original purpose for study participation was to longitudinally investigate the effect of physical exercise on anxiety symptoms over time. STAI assesses both symptoms associated to situations or events and symptoms associated to more stable personal traits. Given the intent to capture longstanding traits, STAI is less suitable to detect change than BAI (Julian 2011). Another limitation is the lack of a non-clinical control group for comparison of measured variables. However, we did perform analyses comparing cognitive function in patients with anxiety disorder, to normed populations. Study participants were recruited on a voluntary basis to take part in an exercise intervention study, which might have produced a selection bias regarding cognitive functioning. Sample size calculations for the original longitudinal RCT study were based on the effect of exercise interventions on symptoms of anxiety and depression [55]. These included two intervention groups and one control group. Specific sample size calculations for the current study, assessing baseline anxiety severity and cognitive functions, were not performed. However, the current study pooled the three groups of patients (two intervention groups and one control group), which should increase the statistical power. The order in which cognitive tests were performed could also affect the test results, given that the WAIS-IV tests were consistently performed before the D-KEFS test. One might speculate that patients with more severe anxiety might experience greater fatigue towards the end of the test procedure (which took approximately 35–45 min) than patients with milder anxiety. However, the last test was design fluency, which showed no association with anxiety severity. The observed association might also be influenced by unmeasured variables affecting both anxiety severity and cognitive function.

## Conclusions and implications

In primary care patients with anxiety (PD, GAD and anxiety not otherwise specified) anxiety severity is negatively associated with EFs related to WM after adjustment of major depression. The current study has implications for the understanding of executive behavioural control in primary care patients with the above-specified anxiety disorder. Characterization of cognitive function in patients with anxiety may facilitate the development of more individualized treatment strategies, that could include interventions to improve specific cognitive domains when indicated. Such individualized strategies could have clinical implications for treatment compliance, symptom reduction, coping mechanisms, as well as overall daily functioning for primary care patients with anxiety disorders. For example, EF may predict treatment response to cognitive behaviour therapy in anxious older adults [56]. Moreover, research on EF may advance the understanding of the psychopathology of anxiety and identify vulnerability factors. Research has shown that EF impairments can elevate the impact of repetitive negative thoughts (including worry and rumination) on the development of anxiety disorder [57]. The potential impact of physical exercise on anxiety severity and tests of cognitive performance in primary care patients with anxiety disorders will be presented in a future publication.

## Supplementary Information

**Additional file 1. **Frequency distribution of performance scores oncognitive tests. Frequency distribution of performance scores on WAIS-IV block design, digitspan and matrix reasoning tests and D-KEFS design fluency test showing totalcorrect designs and total attempted designs subtests, among primary carepatients with anxiety disorders. The black curve represents a normalapproximation curve, for comparison.

**Additional file 2. ** Characteristics of study participants with anxiety disorders with or without ongoing psychotropic medication. Characteristics of study participants with anxiety disorders with or without ongoing antidepressant and/or psycholeptic/anitiepilepticmedication.

**Additional file 3. **Characteristics of study participants with anxiety disorders by anxiety severity category. Characteristics of study participants with anxiety disorders by anxiety severity category.

**Additional file 4.** Performance scores on cognitive testscompared to a normed population. Performancescores on block design, digit-span and matrix reasoning tests (WAIS-IV) and design fluency (D-KEFS) for all patients with anxiety, patients with minimal/mild anxiety and patients with moderate/severe anxiety compared to a normed population.

## Data Availability

Data generated for the current study is not publicly available for ethical reasons but data on group level are available from the corresponding author on reasonable request.

## Abbreviations

BAI: Beck Anxiety Inventory
D-KEFS: Delis-Kaplan Executive Function System
EF: Executive Function
GAD: Generalized Anxiety Disorder
MADRS-S: Montgomery Åsberg Depression Rating Scale
M.I.N.I: Mini International Neuropsychiatric Interview
OCD: Obsessive Compulsive Disorder
PD: Panic Disorder
PTSD: Post Traumatic Stress Disorder
SAD: Social Anxiety Disorder
SD: Standard Deviation
STAI: the State-Trait Anxiety Inventory
WAIS-IV: Wechsler Adult Intelligence Scale IV
WM: Working Memory
