# Supplementary material for: Anxiety severity and cognitive function in primary care patients with anxiety disorder: a cross-sectional study
Source: BMC Psychiatry. 2021 Dec 9;21:617. doi: 10.1186/s12888-021-03618-z (PMC8662874; doi:10.1186/s12888-021-03618-z)
Supplement: Supplementary file 1 — Additional file 1. Frequency distribution of performance scores oncognitive tests. Frequency distribution of performance scores on WAIS-IV block design, digitspan and matrix reasoning tests and D-KEFS design fluency test showing totalcorrect designs and total attempted designs subtests, among primary carepatients with anxiety disorders. The black curve represents a normalapproximation curve, for comparison. [file 12888_2021_3618_MOESM1_ESM.pdf]

Additional file 1. Frequency distribution of performance scores on cognitive tests.

Frequency distribution of performance scores on WAIS-IV block design, digit span and matrix reasoning tests and D-KEFS design fluency test showing total correct designs and total attempted designs subtests, among primary care patients with anxiety disorders. The black curve represents a normal approximation curve, for comparison.

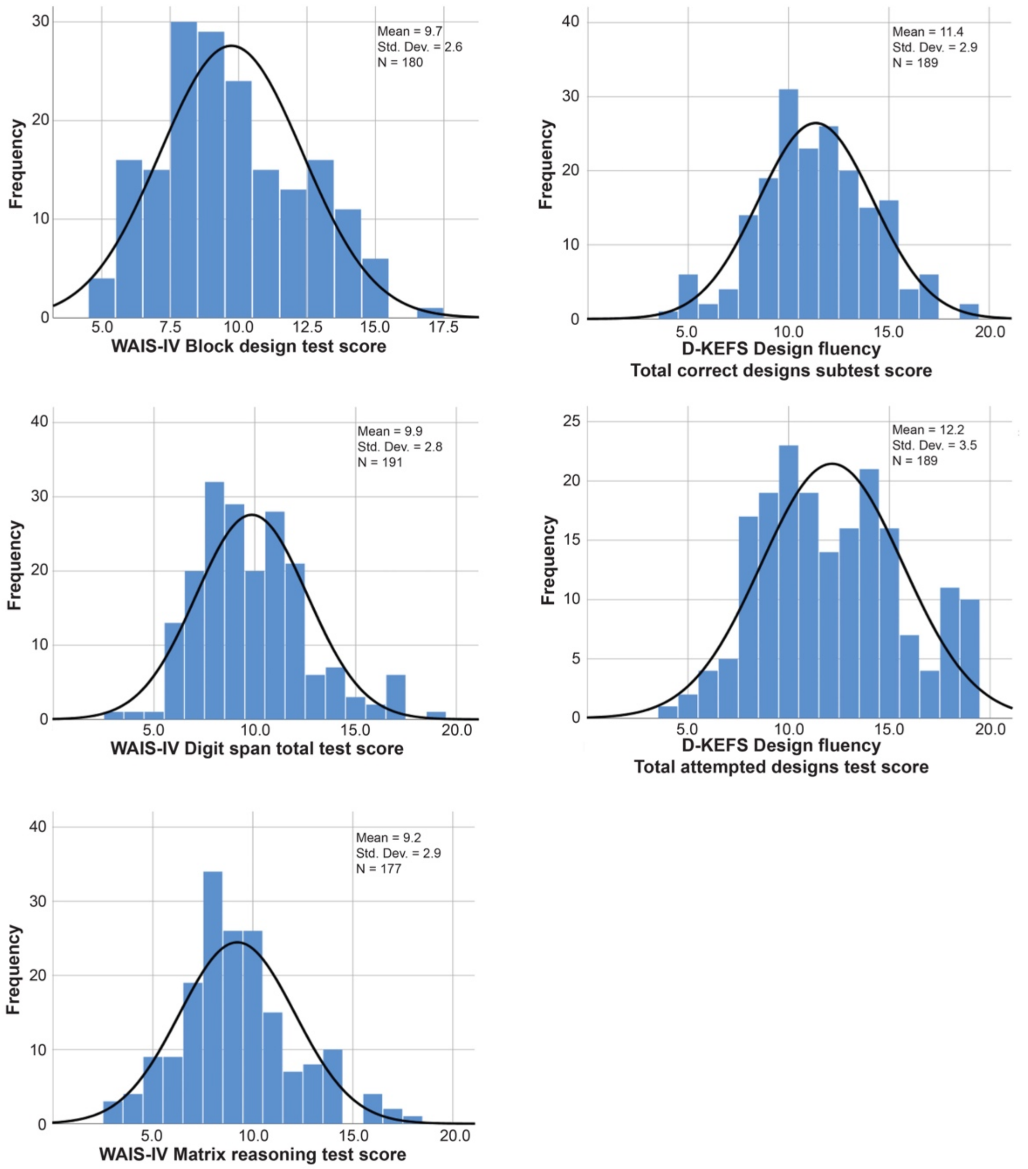

WAIS: Wechsler Adult Intelligence Scale; D-KEFS: Delis–Kaplan Executive Function System; Std.Dev.: standard deviation; N: numbers
