# Supplementary material for: Anxiety severity and cognitive function in primary care patients with anxiety disorder: a cross-sectional study
Source: BMC Psychiatry. 2021 Dec 9;21:617. doi: 10.1186/s12888-021-03618-z (PMC8662874; doi:10.1186/s12888-021-03618-z)
Supplement: Supplementary file 2 — Additional file 2. Characteristics of study participants with anxiety disorders with or without ongoing psychotropic medication. Characteristics of study participants with anxiety disorders with or without ongoing antidepressant and/or psycholeptic/anitiepilepticmedication. [file 12888_2021_3618_MOESM2_ESM.pdf]

Additional file 2. Characteristics of study participants with anxiety disorders with or without ongoing antidepressant and/or psycholeptic/anitiepileptic medication.

	No medication		Medication		
Variables	Mean [SD] or N (%)	N	Mean [SD] or N (%)	N	p-value
Age (years)	36.7 [11.1]	63	39.7 [12.6]	126	0.15
Men	24 (38.1)	63	35 (27.8)	126	0.15
Education above high school	35 (55.6)	63	69 (55.6)	124	0.99
Smoking	5 (8.1)	62	33 (26.2)	126	**0.04**
Psychotropic medication					
Antidepressants[a]			101 (80.2)	126	
Psycholeptics/anitiepileptics[b]			56 (44.4)	126	
Unmarried	41 (67.2)	61	90 (72.6)	124	
Married	20 (32.8)	61	34 (27.4)	124	
**Rating Scales:**					
BAI (score)	24.2 [12.7.8]	63	25.2 [12.7]	126	0.59
MADRS-S (score)	20.0 [8.3]	63	22.5 [8.0]	126	**0.044**
**WAIS-IV test scores:**					
Block design	9.5 [3.0]	57	9.3 [3.2]	120	0.80
Digit span total	9.9 [3.1]	63	9.8 [2.6]	125	0.97
Digit span forward	9.1 [3.5]	57	9.0 [3.3]	120	0.91
Digit span backward	9.7 [3.1]	57	9.9 [2.5]	120	0.36
Digit span sequencing	9.5 [3.1]	57	9.4 [2.7]	120	0.84
Matrix reasoning	9.7 [2.8]	56	9.1 [2.9]	118	0.26
**D-KEFS design fluency test scores:**					
Total correct designs	11.4 [3.0]	63	11.4 [2.8]	123	0.97
Correct designs condition 1	10.4 [3.1]	63	10.7 [2.6]	123	0.46
Correct designs condition 2	10.4 [2.8]	63	10.5 [2.7]	123	0.88
Correct designs condition 3	11.7 [2.6]	63	11.3 [2.8]	123	0.55
Total attempted designs	12.1 [3.5]	63	12.2 [3.5]	123	0.76
**Anxiety diagnoses:**					
Panic disorder	36 (58.1)	62	64 (50.8)	126	0.35
Generalized anxiety disorder	34 (54.8)	62	73 (57.9)	126	0.69
Mixed anxiety and depression	4 (6.5)	62	8 (6.3)	126	0.98
Anxiety (Unspecified)	7 (11.3)	62	12 (9.5)	126	0.71
**Comorbidities:**					
Major depression	22 (35.5)	62	56 (44.4)	126	0.24
Suicidality	9 (14.5)	62	34 (27.0)	126	0.056
Social phobia	23 (37.1)	62	53 (42.1)	126	0.51
Agoraphobia	16 (25.8)	62	48 (38.1)	126	0.095
Post-traumatic stress disorder	9 (14.5)	62	17 (13.5)	126	0.85
Alcohol use disorder	2 (3.2)	62	17 (13.5)	126	**0.028**
Substance use	0	62	4 (3.2)	126	0.16
Personality disorder (Antisocial)	6 (9.7)	62	13 (10.3)	126	0.89
Obsessive compulsive disorder	10 (16.1)	62	10 (7.9)	126	0.087
Bulimia Nervosa	5 (8.1)	62	7 (5.6)	126	0.51

[a] ATC N06

[b] ATC N03 and N05

Values are given as means and standard deviations (SD) for continuous variables and frequencies with percentages for categorical variables along with the total number (N) of participants included in each analysis. P-value was based on χ^2-test and Mann-Whitney U-test comparing patients with Minimal/Mild (BAI 0-16) vs Moderate/Severe anxiety (BAI 17-63). Statistically significant results ($p<0.05$) indicated in bold.

D-KEFS: Delis–Kaplan Executive Function System; MADRS-S: Montgomery Åsberg Depression Rating Scale Self-rated; WAIS: Wechsler Adult Intelligence Scale.
