# Supplementary material for: Anxiety severity and cognitive function in primary care patients with anxiety disorder: a cross-sectional study
Source: BMC Psychiatry. 2021 Dec 9;21:617. doi: 10.1186/s12888-021-03618-z (PMC8662874; doi:10.1186/s12888-021-03618-z)
Supplement: Supplementary file 3 — Additional file 3. Characteristics of study participants with anxiety disorders by anxiety severity category. Characteristics of study participants with anxiety disorders by anxiety severity category. [file 12888_2021_3618_MOESM3_ESM.pdf]

Additional file 3. Characteristics of study participants with anxiety disorders by anxiety severity category.

	Minimal/Mild anxiety		Moderate/Severe anxiety		
Variables	Mean [SD] or N (%)	N	Mean [SD] or N (%)	N	p-value
Age (years)	37.8 [12.3]	61	39.2 [12.2]	128	0.44
Men	28 (45.9)	61	31 (24.8)	128	**0.003**
Education above high school	35 (57.4)	61	69 (54.8)	126	0.74
Smoking	8 (13.1)	61	30 (23.6)	127	0.093
Psychotropic medication	39 (63.9)	61	87 (68.0)	128	0.58
Antidepressants[a]	32 (52.5)	61	69 (53.9)	128	0.85
Psycholeptics/anitiepileptics[b]	14 (23.0)	61	43 (33.6)	128	0.14
Unmarried	38 (63.3)	60	93 (74.4)	125	
Married	22 (36.7)	60	32 (25.6)	125	
**Rating Scales:**					
BAI (score)	11.0 [3.8]	61	31.4 [9.7]	128	**<0.001**
MADRS-S (score)	16.8 [7.9]	61	24.0 [7.2]	128	**<0.001**
**WAIS-IV test scores:**					
Block design	10.0 [2.9]	57	9.1 [3.2]	120	0.095
Digit span total	10.5 [3.0]	61	9.5 [2.6]	127	**0.016**
Digit span forward	9.7 [3.5]	57	8.7 [3.3]	120	**0.042**
Digit span backward	10.6 [2.9]	57	9.5 [2.6]	120	**0.030**
Digit span sequencing	9.9 [2.9]	57	9.2 [2.8]	120	0.085
Matrix reasoning	9.8 [3.1]	55	9.1 [2.7]	119	0.14
**D-KEFS design fluency test scores:**					
Total correct designs	11.6 [2.5]	60	11.3 [3.0]	126	0.28
Correct designs condition 1	10.7 [2.6]	60	10.5 [2.9]	126	0.65
Correct designs condition 2	10.8 [2.7]	60	10.3 [2.7]	126	0.19
Correct designs condition 3	11.6 [2.7]	60	11.3 [2.7]	126	0.44
Total attempted designs	12.2 [3.1]	60	12.2 [3.7]	126	0.89
**Anxiety diagnoses:**					
Panic disorder	20 (32.8)	61	80 (63.0)	127	**<0.001**
Generalized anxiety disorder	34 (55.7)	61	73 (57.5)	127	0.82
Mixed anxiety and depression	8 (13.1)	61	4 (3.1)	127	**0.009**
Anxiety (Unspecified)	9 (14.8)	61	10 (7.9)	127	0.14
**Comorbidities:**					
Major depression	16 (26.2)	61	62 (48.8)	127	**0.003**
Suicidality	12 (19.7)	61	31 (24.4)	127	0.47
Social phobia	21 (34.4)	61	55 (43.3)	127	0.25
Agoraphobia	10 (16.4)	61	54 (42.5)	127	**<0.001**
Post-traumatic stress disorder	2 (3.3)	61	24 (18.9)	127	**0.004**
Alcohol use disorder	7 (11.5)	61	12 (9.4)	127	0.67
Substance use	1 (1.6)	61	3 (2.4)	127	0.75
Personality disorder (Antisocial)	4 (6.6)	61	15 (11.8)	127	0.26
Obsessive compulsive disorder	5 (8.2)	61	15 (11.8)	127	0.45
Bulimia Nervosa	3 (4.9)	61	9 (7.1)	127	0.57

[a] ATC N06
[b] ATC N03 and N05
Values are given as means and standard deviations (SD) for continuous variables and frequencies with percentages for categorical variables along with the total number (N) of participants included in each analysis. P-value was based on χ^2-test and Mann-Whitney U-test comparing patients with Minimal/Mild (BAI 0-16) vs Moderate/Severe anxiety (BAI 17-63). Statistically significant results ($p<0.05$) indicated in bold.
D-KEFS: Delis–Kaplan Executive Function System; MADRS-S: Montgomery Åsberg Depression Rating Scale Self-rated; WAIS: Wechsler Adult Intelligence Scale.
