# Supplementary material for: Anxiety severity and cognitive function in primary care patients with anxiety disorder: a cross-sectional study
Source: BMC Psychiatry. 2021 Dec 9;21:617. doi: 10.1186/s12888-021-03618-z (PMC8662874; doi:10.1186/s12888-021-03618-z)
Supplement: Supplementary file 4 — Additional file 4. Performance scores on cognitive testscompared to a normed population. Performancescores on block design, digit-span and matrix reasoning tests (WAIS-IV) and design fluency (D-KEFS) for all patients with anxiety, patients with minimal/mild anxiety and patients with moderate/severe anxiety compared to a normed population. [file 12888_2021_3618_MOESM4_ESM.pdf]

Additional file 4. Performance scores on cognitive tests in patients with anxiety disorders compared to a normed population.

Cognitive function test	Mean	SD	N-1	t*	Wilcoxon p
WAIS IV					
Block design	9.37	3.11	176	-2.71	**0.049**
Digit span total	9.85	2.77	187	-0.74	0.11
Digit span forward	9.03	3.38	176	-3.83	**<0.001**
Digit span backward	9.83	2.72	176	-0.83	0.15
Digit span sequencing	9.39	2.83	176	-2.87	**0.001**
Matrix reasoning	9.27	2.88	173	-3.34	**<0.001**
D-KEFS design fluency					
Total correct designs	11.39	2.85	185	6.65	**<0.001**
Correct designs condition 1	10.60	2.80	185	2.91	**0.006**
Correct designs condition 2	10.47	2.70	185	2.39	**0.036**
Correct designs condition 3	11.43	2.74	185	7.12	**<0.001**
Total attempted designs	12.18	3.50	185	8.50	**<0.001**

Performance scores on block design, digit-span and matrix reasoning tests (WAIS-IV) and design fluency (D-KEFS) for patients with anxiety disorder compared to a normed population.
* normed mean for the population is 10 (SD3) for WAIS IV and D-KEFS.
SD: standard deviation; N-1: degrees of freedom; t: t-values from the $F(df_{regression},df_{residual})=F_{regression}$ equation.
WAIS: Wechsler Adult Intelligence Scale; D-KEFS: Delis–Kaplan Executive Function System
